# Impact of the Distance from the Stent Edge to the Residual Plaque on Edge Restenosis following Everolimus-Eluting Stent Implantation

**DOI:** 10.1371/journal.pone.0121079

**Published:** 2015-03-16

**Authors:** Masao Takahashi, Susumu Miyazaki, Masahiro Myojo, Daigo Sawaki, Hiroshi Iwata, Arihiro Kiyosue, Yasutomi Higashikuni, Tomofumi Tanaka, Daishi Fujita, Jiro Ando, Hideo Fujita, Yasunobu Hirata, Issei Komuro

**Affiliations:** 1 Department of Cardiovascular Medicine, Graduate School of Medicine, the University of Tokyo, Tokyo, Japan; 2 Department of Medical Engineering, the University of Tokyo, Tokyo, Japan; 3 Tokyo Teishin Hospital, Tokyo, Japan; North Carolina A&T State University, UNITED STATES

## Abstract

**Objectives:**

This study aimed to assess the relation between stent edge restenosis (SER) and the distance from the stent edge to the residual plaque using quantitative intravascular ultrasound.

**Background:**

Although percutaneous coronary intervention with drug-eluting stents has improved SER rates, determining an appropriate stent edge landing zone can be challenging in cases of diffuse plaque lesions. It is known that edge vascular response can occur within 2 mm from the edge of a bare metal stent, but the distance to the adjacent plaque has not been evaluated for drug-eluting stents.

**Methods:**

A total of 97 proximal residual plaque lesions (plaque burden [PB] >40%) treated with everolimus-eluting stents were retrospectively evaluated to determine the distance from the stent edge to the residual plaque.

**Results:**

The SER group had significantly higher PB (59.1 ± 6.1% vs. 51.9 ± 9.1% for non-SER; P = 0.04). Higher PB was associated with SER, with the cutoff value of 54.74% determined using receiver operating characteristic (ROC) curve analysis. At this cutoff value of PB, the distance from the stent edge to the lesion was significantly associated with SER (odds ratio = 2.05, P = 0.035). The corresponding area under the ROC curve was 0.725, and the cutoff distance value for predicting SER was 1.0 mm.

**Conclusion:**

An interval less than 1 mm from the proximal stent edge to the nearest point with the determined PB cutoff value of 54.74% was significantly associated with SER in patients with residual plaque lesions.

## Introduction

Accumulating evidence suggests that utilization of drug-eluting stents (DES) in percutaneous coronary intervention (PCI) leads to a lower incidence of in-stent restenosis (ISR) [1,2]. As DESs continue to evolve, clinical outcomes of their usage improve. In particular, second-generation everolimus-eluting stents (EES) are superior to first-generation DES in terms of both safety and effectiveness [3]. However, it is difficult to determine the optimal landing point for the stent edge in the case of diffuse plaque lesions that occasionally occur in clinical practice. A previous study used intravascular ultrasound (IVUS), a technique superior to angiography for assessing vessel size and lesion severity, to reveal that higher residual plaque area and stent overexpansion are associated with stent edge restenosis (SER) in the first-generation DES implantation [4]. Moreover, another study showed a positive correlation between reference plaque burden (PB) and SER [5]. Although a complete coverage of the plaque lesion is an effective strategy for preventing SER, it can result in multiple long stents (the so-called “full metal jacket”), leading to a high incidence of periprocedural myocardial infarction [6] and increased risk of late thrombosis [7]. This makes PCI with DES controversial in the case of long lesions [8]. Furthermore, edge vascular response was observed within 2 mm from bare metal stent (BMS) edge [9], which implies that the distance to the next plaque is an important factor for SER. In diffuse plaque lesions, it is difficult to determine not only appropriate plaque-free landing zones, but also the optimal distance from the stent edge to the next plaque for stent implantation. Therefore, the aim of the present study was to assess the relation between SER and the distance from the stent edge to the proximal significant residual plaque in patients undergoing EES implantation with residual plaque in a proximal lesion.

## Materials and Methods

### Study design and patient population

**Fig 1:**
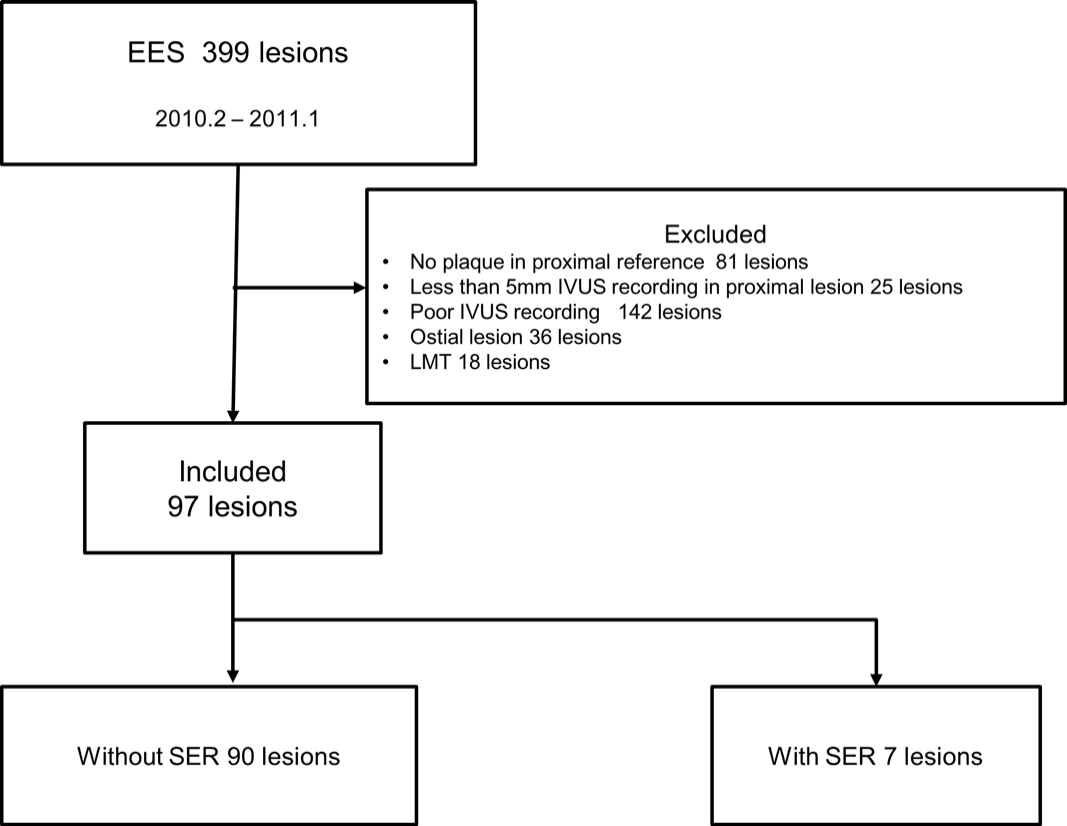
Study flow chart. EES: everolimus-eluting stent; IVUS: Intravascular ultrasound; LMT: left main trunk; SER: stent edge restenosis;

The study population was collected from among PCI patients who provided written informed consent for follow-up angiography. We retrospectively selected patients implanted with EES (Xience V: Abbot Vascular, Santa Clara CA, USA, and Promus: Boston Scientific, Natick MA, USA) at the University of Tokyo Hospital between February 2010 and January 2011. Total 399 lesions (273 patients) were enrolled into this study. Inclusion criteria for this study were as follows: performing elective IVUS-guided PCI and residual PB > 40% in the proximal reference vessel on IVUS. According to a previous report, the residual uncovered PB was about 40% [10]. Another study showed that proximal stent edge dissection was observed for PB of 56.8 ± 11.3% but did not occur for PB of 35.5 ±14.5% [11]. Based on these results, we included patients who had PB > 40% in the proximal reference vessel on IVUS examination. The exclusion criteria were PCI for acute myocardial infarction, the presence of lesions in the left main trunk and the presence of saphenous vein graft. Patients with no available IVUS images of the proximal reference segment because of either ostial lesion or poor recording quality were also excluded. As a result, a total of 97 proximal reference segments (86 patients) were included in this study (Fig. 1).

### Ethics

This study was retrospective chart review and the medical record data were anonymized prior to data access and analysis. This observational study, which adhered to the principles of the Declaration of Helsinki, was approved by the institutional ethical committee of the University of Tokyo (#2650, October 26, 2009). Written informed consent was not taken because the institutional ethical committee states that for this analysis this is not due.

### Data samplings and definitions

Revascularization was defined as ischemia-driven if the angiographic diameter stenosis was more than 75%. Initially, we attempted to cover the plaque to the highest possible extent. However, since residual plaque was present in all cases, the stent was placed in the region with a lower PB and/or higher lumen area in the proximal reference vessel as assessed using IVUS. The final post-stenting IVUS imaging was performed after intracoronary administration of 0.2 mg nitroglycerin using automated motorized pullback (0.5 mm/s) and a commercial scanner (Boston Scientific Scimed, Minneapolis MN, USA) consisting of a rotating 40-MHz transducer within a 3.2 F imaging sheath. All IVUS images were reviewed and their quantitative parameters were evaluated using commercially available software (echoPlaque version 3.0, Indec Systems Incorporated, Santa Clara CA, USA) by an independent, experienced analyst who was blinded to clinical and angiographic lesion characteristics. The stent cross-sectional area and external elastic membrane area of the in-stent segment were evaluated at 0.5-mm intervals using 2-dimensional planimetry. The lumen area and the external elastic membrane area of the proximal reference segment were also evaluated within 5 mm at 0.5-mm intervals. The PB in all areas was calculated as plaque area/external elastic membrane area × 100 (%).

All clinical data regarding baseline demographics, concomitant diseases, lesion characteristics, procedures, and devices were prospectively recorded in the institutional database and retrospectively reviewed by experienced data managers. The mean follow-up period was 231.8 ± 111.1 days for all patients. SER was defined as diameter stenosis >50% located ≤5 mm from the stent margin, but not within the stent [12].

A patient was defined as having diabetes if their hemoglobin A1c was >6.5% and/or they were receiving anti-diabetic agents, including insulin regardless of fasting glucose levels [13]. Patients were diagnosed with hypertension if their systolic/diastolic blood pressure on admission was >140/90 mmHg [14] or if they were under treatment with antihypertensive agents. Similarly, patients were diagnosed as having dyslipidemia if they fulfilled the criteria established by the Japan Atherosclerosis Society and of other guidelines [15]. We also evaluated the following laboratory parameters (hemoglobin A1c, brain natriuretic peptide) and estimated glomerular filtration rate (eGFR) at baseline in all patients. The eGFR was calculated based on the Japanese equation that applies serum Cr level, age, and gender as follows:

$$\text{eGFR}\left(\text{m1/min/1}{\text{.73 m}}^{\text{2}}\right)=194\times {\text{Cr}}^{\text{-1}.094}\times {\text{age}}^{\text{-0}.287}\left(\times 0.739\text{for female patients}\right)$$

### Statistical analysis

Baseline characteristics by patient and by lesion are reported as number and proportion or mean ± standard deviation, as appropriate. The Student *t* test or Mann-Whitney test was used to compare two groups. Univariate logistic regression analysis was used to assess the association with SER. Multivariate logistic analysis was conducted including the parameters that showed significant association with SER in univariate analysis, and known risk factors for coronary artery disease. Receiver operating characteristic (ROC) analysis was performed to calculate the area under the curve (AUC) and define cutoff points. All statistical analyses were performed using JMP software version 9.0 (SAS Institute Inc., Cary, NC, USA). All analyses were two-sided, and a P-value of less than 0.05 was considered statistically significant.

## Results

### Patient characteristics

The clinical characteristics of all lesions (399) and residual plaque lesions (97) are shown in Table 1. In patients with residual plaque lesions, proximal SER developed in 7 cases (7.2%), as determined with angiography. All patients were receiving dual anti-platelet therapy, and stent thrombosis did not occur in this study group. There were no significant differences in clinical characteristics between all patients and those with residual plaque lesions. Furthermore, no such differences were detected between the patients with and without proximal SER. PCI device data and post-stenting IVUS findings for all 97 lesions are summarized in Table 2. The deployed stent diameter, length, and inflation pressure, as well as the post-balloon diameter and inflation pressure were similar in both groups. The results of quantitative post-stenting IVUS of the proximal side region revealed that the lumen cross-section area and minimum lumen area were significantly lower in the patients with SER than in those without SER (P = 0.023). Furthermore, the PB was also significantly higher in patients with SER than in those without SER (P = 0.044), whereas plaque and vessel areas did not show significant differences between the two groups. Similarly, the IVUS parameters of the stent side region revealed no significant differences. In agreement with the presence of a significant difference in the lumen area, the ratio of the stent proximal reference side area to the lumen area was relatively high in patients with SER. There was neither evidence of edge dissection-related SER nor under-expansion of stent in the study groups.

**Table 1:** Background of patients in this study.

variable		all	%	Residual plaque	%	P Value	without SER	%	With SER	%	P value
N (lesion)		274 (399)		86 (97)			81 (90)		7 (7)		
Age		67.5±9.7		66.5±9.2		0.426	66.4±9.7		68.0±8.5		0.330
Men		213	77.7	63	73.3	0.393	58	71.6	7	100.0	0.098
hypertension		217	79.2	71	82.6	0.498	66	81.5	7	100.0	0.226
Diabetes Mellitus		140	51.1	47	54.7	0.566	44	54.3	5	71.4	0.359
Dyslipidemia		195	71.2	63	73.3	0.709	59	72.8	5	71.4	0.965
Smoking		175	63.9	49	57.0	0.251	45	55.6	4	57.1	0.936
Familial History		97	35.4	30	34.9	0.930	28	34.6	4	57.1	0.288
Chronic Kidney disease		117	42.7	31	36.0	0.275	29	35.8	3	42.9	0.613
pPCI		112	40.9	30	34.9	0.393	28	34.6	2	28.6	0.844
pCABG		35	12.8	5	5.8	0.073	5	6.2	0	0.0	-
HbA1c (%)		6.5±0.9		6.4±0.9		0.368	6.5±1.0		6.4±0.9		0.876
BNP (pg/mL)		164.6±371.7		164.2±483.5		0.993	151.7±472.9		133.0±150.6		0.917
eGFR (ml/min/1.73 m^2^)		57.7±23.4		60.5±22.8		0.338	62.4±23.3		53.9±17.2		0.521
target artery	LMT	18	4.5	-			-		-		
	LAD	186	46.6	45	46.4		40		5		
	LCx	92	23.1	25	25.8		24		1		
	RCA	113	28.3	27	27.8		26		1		
DAPT		274	100.0	97	100.0	-	81		7		-
follow-up period (day)		231.8±111.1		213.1±68.1		0.283	210.1±66.1		251.6±86.2		0.781
SER		16	4.0	7	7.2	0.179					

pPCI: previous history of percutaneous coronary intervention.

pCABG: previous history of coronary artery bypass grafting.

eGFR: estimated glomerular filtration rate.

LMT: left main trunk.

LAD: left anterior descending artery.

LCx: left circumflex artery.

RCA: right coronary artery.

DAPT: dual antiplatelet therapy.

**Table 2:** Procedural characteristics and post-stenting intravascular ultrasound (IVUS) findings.

Variable	SER (+)	SER (-)	P value
**N**	7	90	
**Stent diameter (mm)**	2.79±0.2	2.99±0.4	0.163
**Stent length (mm)**	21.0±5.5	18.2±6.3	0.254
**Stent inflation pressure (atm)**	9.4±1.9	10.9±2.1	0.067
**Post-balloon diameter (mm)**	2.93±0.28	3.18±0.44	0.139
**Maximum inflation pressure (atm)**	16.0±1.6	15.8±3.0	0.828
**Post-stenting IVUS findings**			
**stent side**			
**Lumen area (mm^2^)**	6.5±1.7	7.1±2.0	0.447
**Minimum lumen area (mm^2^)**	6.0±1.6	6.4±1.8	0.582
**Vessel area (mm^2^)**	13.9±3.7	14.8±3.7	0.568
**Plaque area (mm^2^)**	7.4±2.1	7.6±2.4	0.741
**proximal side**			
**Lumen area (mm^2^)**	4.9±1.1	6.9±2.3	*0.023*
**Minimum lumen area (mm^2^)**	3.9±0.9	5.8±2.0	*0.013*
**Vessel area (mm^2^)**	11.9±2.2	14.7±3.9	0.069
**Plaque area (mm^2^)**	7.1±1.6	7.8±2.5	0.463
**Plaque burden (%)**	59.1±6.1	51.9±9.1	*0.044*
**Stent/Lumen ratio**	1.13±0.10	1.03±0.11	*0.021*
**Stent/Vessel ratio**	0.72±0.06	0.70±0.08	0.599

SER: stent edge restenosis.

Data are presented as mean ± SD.

Stent/Lumen ratio is the ratio of stent area to mean lumen area of the proximal reference.

Stent/Vessel ratio is the ratio of stent area to mean vessel area of the proximal reference.

P values suggesting statistical significance are shown in italics.

### Proximal lumen area and PB were associated with SER

**Fig 2:**
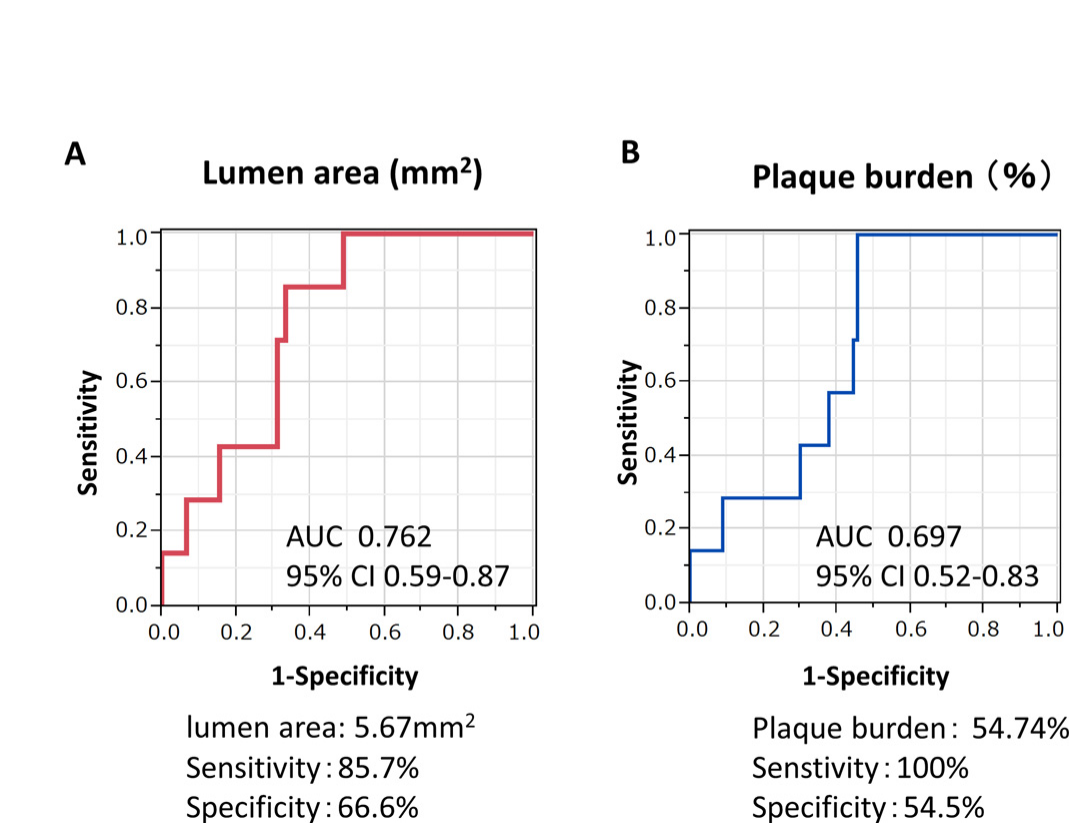
Receiver operating characteristic analysis of lumen area (A) and plaque burden (B) of proximal reference segment. Cutoff values predicting SER are shown below the graphs. AUC: area under curve, CI: confidence interval.

Logistic regression analysis revealed that the lumen area and PB significantly correlated with SER (Table 3). Because the lumen area and PB of the proximal reference segment were associated with proximal SER, optimal cutoff values of both parameters were evaluated (Fig. 2). In patients with proximal residual plaque, the lumen area <5.67 mm^2^ predicted SER with the sensitivity of 85.7% and specificity of 66.6%, whereas PB >54.74% predicted SER with the sensitivity of 100% and specificity of 54.5%.

**Table 3:** Logistic regression analysis for stent edge restenosis.

	odds ratio	95% confidential interval	P value
Lumen area	1.88	1.14–3.86	0.039
PB	0.88	0.77–0.98	0.041

PB: plaque burden.

### Distance from stent edge to residual plaque

**Fig 3:**
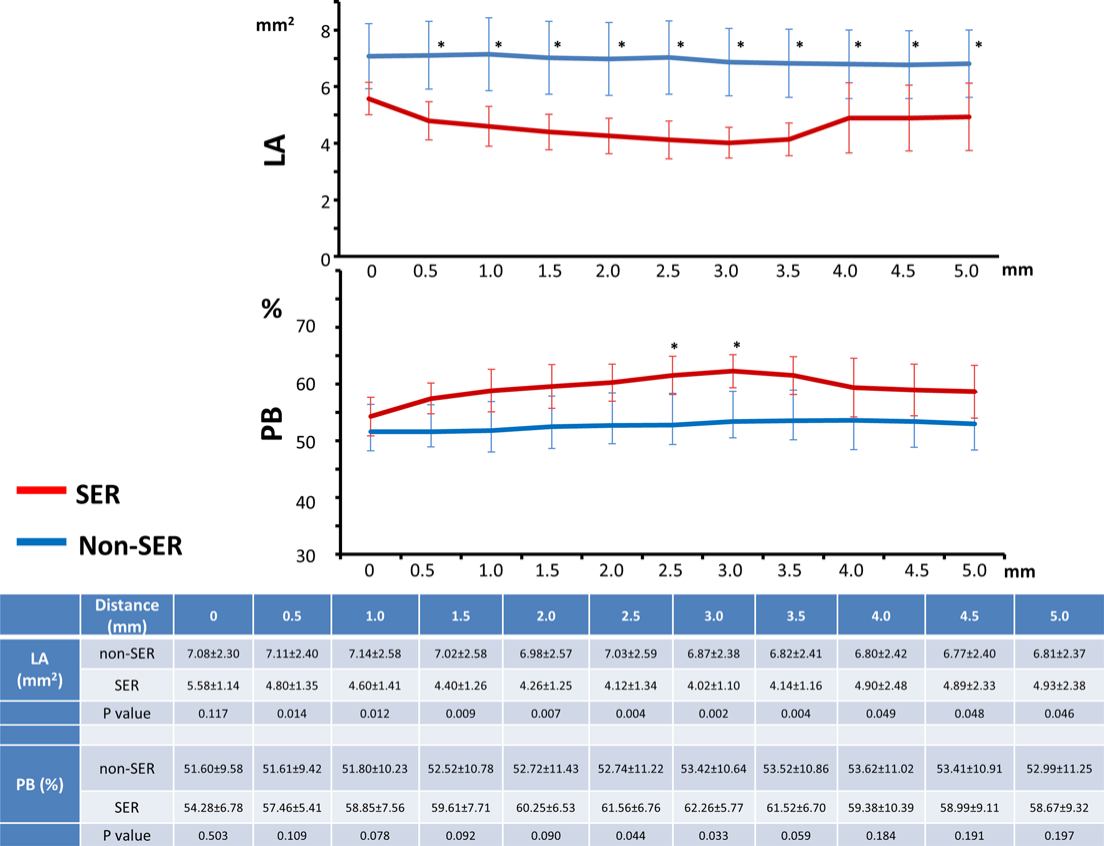
Values of lumen area (LA) and plaque burden (PB) within the 5 mm region proximal to the stent edge determined at 0.5mm intervals. SER: stent edge restenosis. Symbol * shows that there is a statistically significant difference between the SER and non-SER groups.

**Fig 4:**
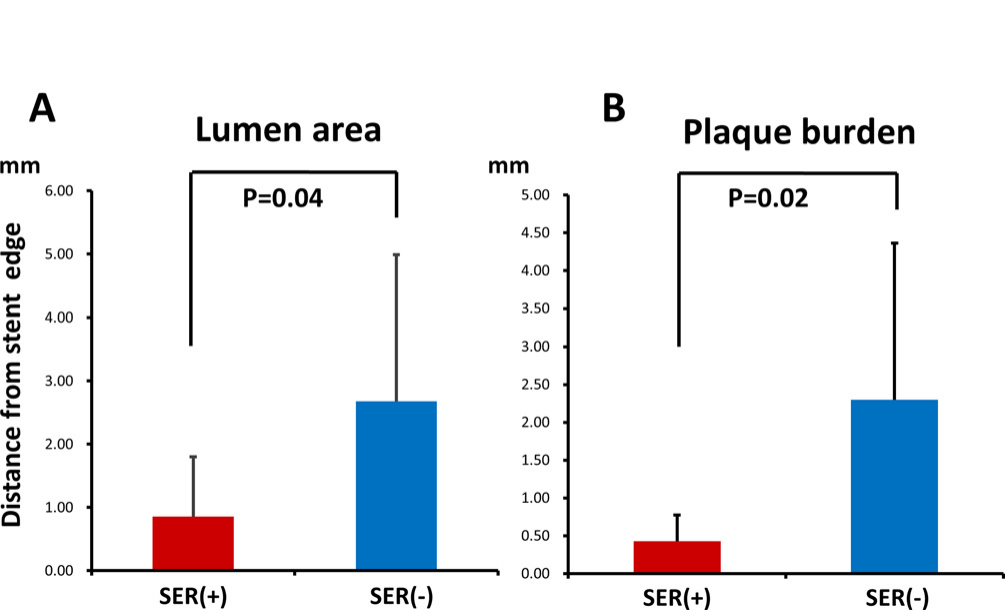
Distances from the stent edge to the nearest points with a lumen area of 5.67 mm^2^ (A) and a PB of 54.74% (B) in patients with and without SER. Lumen area: 0.86 ± 0.94 mm (SER) vs. 2.68 ± 2.31mm (non-SER), P = 0.04; PB: 0.43 ± 0.35 mm (SER) vs. 2.30 ± 2.06 mm (non-SER), P = 0.02.

**Fig 5:**
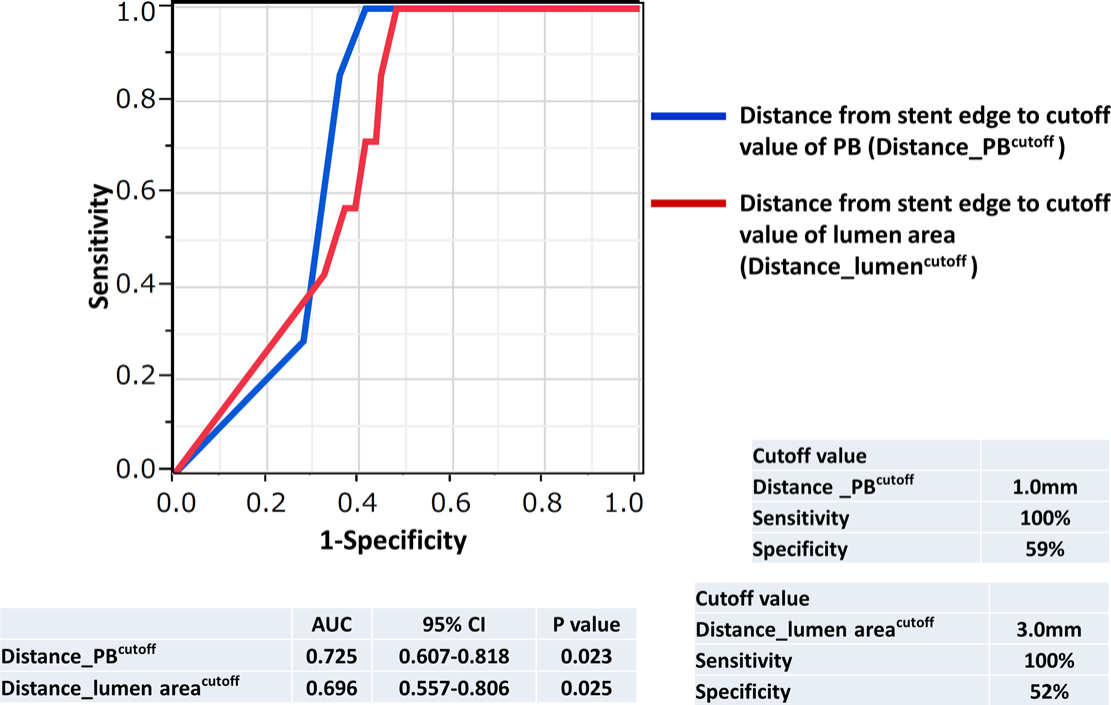
Receiver-operating characteristic analysis of distance from the stent edge to the nearest points with a lumen area cutoff value (Distance_lumen^cutoff^) and a PB cutoff value (Distance_PB^cutoff^) for SER. Statistical parameters of Distance_lumen^cutoff^ and Distance_PB^cutoff^ for predicting SER are shown below the graphs.

The values of the lumen area and PB in the region within 5 mm from the stent edge evaluated at 0.5 mm intervals are presented in Fig. 3. Lumen areas in the SER group were significantly lower at all distances from the stent edge. Moreover, the lumen area of the SER group decreased in the direction from the stent edge toward the proximal reference segment, reaching the lowest value 3.0 mm from the stent edge. In the SER group, PB values were significantly higher 2.5 mm and 3.0 mm from the stent edge than those in the non-SER group. The distances from the stent edge to the nearest point with the lumen area of 5.67 mm^2^ (the cutoff value for the lumen area) and PB of 54.74% (the cutoff value for PB), termed Distance_lumen^cutoff^ and Distance_PB^cutoff^, respectively, were significantly higher in the non-SER group (Distance_lumen^cutoff^: 0.86 ± 0.94 mm (SER) vs. 2.68 ± 2.31 mm (non-SER), P = 0.04; Distance_PB^cutoff^: 0.43 ± 0.35 mm (SER) vs. 2.30 ± 2.06 mm (non-SER), P = 0.02) (Fig. 4). The results of univariate logistic regression analysis indicated that Distance_PB^cutoff^ was a risk factor for SER (odds ratio 2.05, P = 0.035, 95% confidence interval 1.04–7.79), whereas Distance_lumen^cutoff^ was not (odds ratio 1.26, P = 0.294, 95% confidence interval 0.83–2.19). Multivariate logistic regression analysis revealed that Distance_PB^cutoff^ was a risk factor for SER (Table 4). According to the ROC curve analysis, the AUCs of Distance_lumen^cutoff^ and Distance_PB^cutoff^ for SER were 0.696 and 0.725, respectively (Fig. 5). Statistical parameters of Distance_lumencutoff and Distance_PBcutoff for predicting SER are shown below the graphs. The optimal cutoff point of Distance_PB^cutoff^ was found to be 1.0 mm, and it predicted SER with the sensitivity of 100.0% and specificity of 59.2%. The optimal cutoff point of Distance_lumen^cutoff^ was 3.0 mm, and the sensitivity and specificity were 100.0% and 52.2%, respectively. As revealed by AUC analysis, the diagnostic value of the combination of Distance_PB^cutoff^ and Distance_lumen^cutoff^ was significantly higher than that of each of these parameters alone (S1 Fig.). When used in combination, the optimal cutoff values of Distance_lumen^cutoff^ and Distance_PB^cutoff^ were found to be 1.0 mm and 2.5 mm, respectively, and the corresponding sensitivity and specificity were 100.0% and 62%.

**Table 4:** Multivariate Logistic regression analysis for stent edge restenosis.

	odds ratio	95% confidential interval	P value
Age	0.98	0.87–1.09	0.697
Diabetes Mellitus	0.39	0.02–4.93	0.462
Hypertension	0.00004	0.00–2.38	0.161
Dyslipidemia	1.06	0.09–11.33	0.959
Smoking	0.49	0.04–4.18	0.525
Familial History	0.26	0.03–1.79	0.174
eGFR (ml/min/1.73 m^2^)	1.02	0.97–1.07	0.500
BNP (pg/mL)	1.01	0.99–1.01	0.916
HbA1c (%)	1.78	0.48–8.75	0.409
Distance_PB^cutoff^	2.10	1.10–7.97	*0.018*

eGFR: estimated glomerular filtration rate, BNP: brain natriuretic peptide, HbA1c: hemoglobin A1c.

Distance_PB^cutoff^: The distance from stent edge to the nearest point of plaque burden cutoff value (54.74%).

P values suggesting statistical significance are shown in italics.

## Discussion

In the DES era, the incidence of ISR is substantially reduced because the intimal hyperplasia is suppressed by the “eluted drug”. Therefore, more complex lesions such as long diffuse lesions and small vessel lesions are now targeted for revascularization [16], and, consequently, residual plaques are much more common in clinical practice. Even though lumen area and PB could predict SER in the case of patients with diffuse plaque lesions, it is still challenging to unequivocally determine the landing point of the stent edge. The main finding of the present analysis is that patients with SER are characterized by shorter distances from the stent edge to the points where the lumen area and PB reach the cutoff values of 5.67 mm^2^ and 54.74%, respectively. Moreover, we found that the cutoff distance from the stent edge to the point with PB > 54.74% that predicts SER is 1.0 mm. ROC curve analysis showed that a combination of Distance_PB^cutoff^ and Distance_lumen^cutoff^ (distances from the stent edge to the nearest points with cutoff values of the PB and lumen area, respectively) has a higher AUC, and therefore a better predicting power, than the individual parameters. Regardless of whether used alone or in combination, the optimal cutoff value of Distance_PB^cutoff^ was 1.0 mm. In this regard, a previous report showed that PB > 55% predicts proximal SER [5]. Overall, our data are in agreement with this result, although the mentioned study suggested a slightly higher cutoff value for the minimum lumen area that leads to SER (<7.1mm^2^). A possible reason for this difference is the diverse background of the study patients. Moreover, the patients included in the present study had PB of more than 40% in the proximal reference vessel, suggesting that a higher PB might lead to a smaller lumen area. Our data does not mean additional stents or longer stent can generate sufficient results for SER, but adjustment of the stent length according to the residual PB and the distance to PB might prevent the SER.

SER after BMS implantation was mainly attributed to plaque and intimal hyperplasia increase becoming more evident within 2 mm from the stent edge [9]. In the TAXUS trial, integrated IVUS analysis suggested that the change in the proximal segment of plaque area in patients with paclitaxel-eluting stent implantation did not result in a significant difference compared to that in patients with BMS implantation [17]. Furthermore, although IVUS analysis of EES implantation in the SPIRIT III trial showed that vessel size, plaque, and lumen volume index (mm^3^/length) of proximal adjacent segments were quite similar to the corresponding values determined during the follow-up period [18], precise distances from the stent edge to the points with cutoff lumen area and PB were not evaluated. The second generation stent EES may attenuate the edge vascular response. Therefore, the relation between EES edge restenosis and the distance from the stent edge to the residual plaque still needs to be investigated in patients with diffuse plaque lesion. Although a complete coverage of plaque is one of the strategies for prevention of SER, long stenting may lead to a high incidence of side branch occlusion [6] and late thrombosis [7], even using the DES. Moreover, normal-to-normal coverage of the plaque can be performed only for relatively focal plaque lesions. In the case of diffuse lesions, low plaque burden should be considered the main characteristic of the proximal reference segment when determining the optimal stent edge landing point. In this regard, choosing the distance >1.0 mm to the next area with high plaque burden (PB>55%) may contribute to preventing SER. One of the more likely explanations for our findings is that, in contrast to BMS, “eluted drug” which suppress the edge vascular response may be contributing to the interval.

It is also reasonable to maintain some degree of interval from stent edge to next high plaque burden in term of less incidence of stent edge injury due to mismatch between balloon size and lumen area. From that perspective, the adequate stent edge landing point should be determined by using the precise IVUS analysis. Although angiography-guided PCI, which can detect the lesion and treat the stenosis, has an advantage of a lower cost and less cumbersome procedure [19], the optimal landing point of the stent edge could not be determined with this technique. It was reported that maximum necrotic core was most often observed proximally to the minimum lumen area, which led to a larger remodeling index [20]. IVUS can detect relatively high residual plaque at preserved lumen area, and can also identify the 55% PB point in the middle of the lesion. Therefore, IVUS-guided PCI is beneficial for preventing the SER in cases with diffuse plaque lesions.

Accumulating evidence suggests that SER is a multifactorial phenomenon [21]. Higher plaque burden and lower lumen area are known risk factors of SER [4,5]. Although some instances of SER were due to dissection induced by stents and balloon [22–24], IVUS imaging revealed no such cases in the present study. However IVUS (40-MHz) which giving a practical axial spatial resolution of approximately 100–200 μm, may not always detect minor stent edge dissection. These minor dissections can be detected by optical coherence tomography (OCT) which has an ability of higher spatial resolution. Although previous study showed OCT-detected minor dissection may not require additional treatment [25], large-scale, prospective study is necessary to reveal the clinical impact of OCT-detected minor dissection. It was also suggested that the stent design can contribute to the risk of SER [21]. However, this factor did not affect our results since only EES with consistent drug and polymer (Xience and Promus) were utilized. In the SER group, the stent edge lumen area was 5.58 ± 1.14 mm^2^, and the PB was 54.28 ± 6.78%. Of note, this lumen area was not the lowest, whereas PB was not the highest within the 5.0 mm-long proximal reference segment. These results indicate that we attempted to place the stent in the region with a higher lumen area and lower PB in diffuse plaque lesions imaged with IVUS. According to our results, Distance_PB^cutoff^ significantly correlated with SER, but Distance_lumen^cutoff^ did not. There are several possible reasons for this. First, the area of the lumen was diffusely small in the SER group, and therefore Distance_lumen^cutoff^ might not reach statistical significance because of the low variability. Second, since lumen area can be evaluated with angiography, physicians who performed the procedure tended to keep the stent edge away from these narrow regions rather than from areas with higher PB. Narrow lumen areas are immediately evident to the operator even when using IVUS, whereas areas with higher PB can only be detected after processing of the IVUS data.

The present study has some obvious limitations. First, because it presents a retrospective single-center analysis, selection bias may not be entirely excluded. Owing to the stringent inclusion criteria, the number of subjects was relatively small. Hence, a large-scale, multi-center, prospective study is necessary to confirm the results of this report. Second, we did not evaluate the IVUS results during the follow-up period, and therefore the precise mechanism of SER remains unclear. Despite these limitations, our results suggest that the distance from the stent edge to the point with PB > 54.74% is significantly associated with the risk of SER.

## Conclusions

In conclusion, to prevent SER, low plaque burden should be considered the main characteristic of the proximal reference segment when choosing the stent edge landing point. In particular, an interval of less than 1 mm from the proximal stent edge to the nearest point with plaque burden of 54.74% in patients with proximal residual plaque lesion is associated with SER. Adjustment of stent length according to the plaque burden value and the distance might prevent the SER.

## Supporting Information

S1 Fig Receiver-operating characteristic analysis of distance from the stent edge to the nearest point with a PB cutoff value (Distance_PB^cutoff^) in combination with the distance from the stent edge to the nearest point with a lumen area cutoff value (Distance_lumen^cutoff^) for SER. The AUC of combined distances is significantly higher than those of individual components. *P value: compared to that of the combination.
(TIF)
