# Supplementary figures and images for: Impact of the Distance from the Stent Edge to the Residual Plaque on Edge Restenosis following Everolimus-Eluting Stent Implantation
Source: PLoS One. 2015 Mar 16;10(3):e0121079. doi: 10.1371/journal.pone.0121079 (PMC4361545; doi:10.1371/journal.pone.0121079)

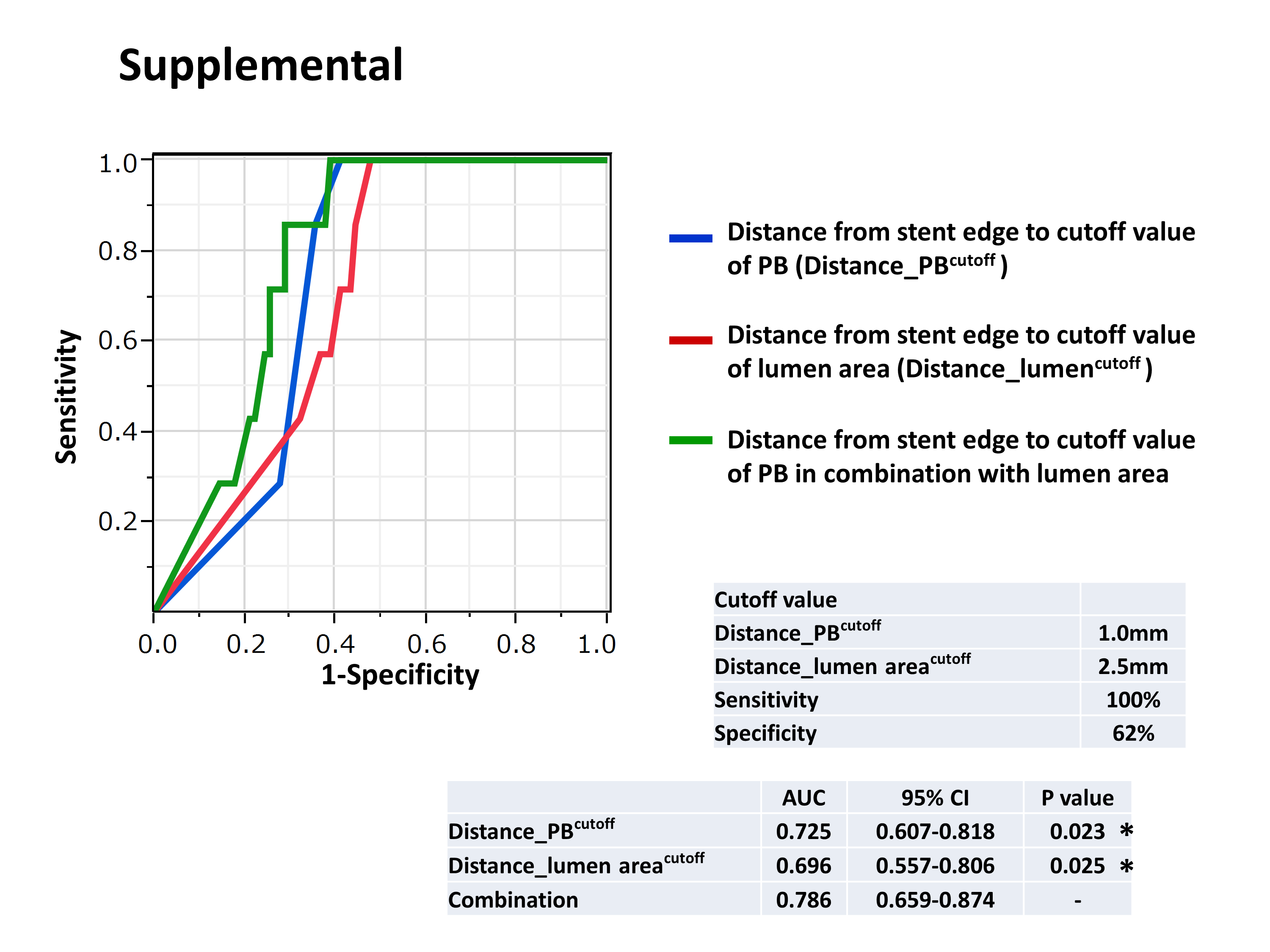

Supplement: S1 Fig — The AUC of combined distances is significantly higher than those of individual components. *P value: compared to that of the combination. (TIF) [file pone.0121079.s001.tif]
